# Reply to DeVore, G.R. Determination of the Same Cardiac Cycle When Analyzing Cine Clips for Use by FetalHQ to Assess Intraobserver and Interobserver Variability. Comment on “Mlodawski et al. Reproducibility Challenges in Fetal Cardiac Function Analysis with 2D Speckle-Tracking Echocardiography: Insights from FetalHQ. *J. Clin. Med.* 2025, *14*, 3301”

**DOI:** 10.3390/jcm15072469

**Published:** 2026-03-24

**Authors:** Jakub Mlodawski, Anna Zmelonek-Znamirowska, Lukasz Pawlik, Marta Mlodawska, Grzegorz Swiercz

**Affiliations:** 1 Collegium Medicum, Jan Kochanowski University in Kielce, 25-516 Kielce, Poland; rjawka@poczta.fm (A.Z.-Z.); marta.mlodawska@ujk.edu.pl (M.M.); grzegorz.swiercz@ujk.edu.pl (G.S.); 2 Clinic of Obstetrics and Gynecology, Provincial Combined Hospital in Kielce, 25-736 Kielce, Poland; 3 Department of Information Systems, Kielce University of Technology, 25-314 Kielce, Poland; lpawlik@tu.kielce.pl

We thank Dr. Greggory R. DeVore for his thoughtful and detailed comments on our article “Reproducibility Challenges in Fetal Cardiac Function Analysis with 2D Speckle-Tracking Echocardiography: Insights from FetalHQ” [1]. We are genuinely grateful that someone so closely involved in the development of FetalHQ has taken the time to engage with our work.

First, we would like to clarify precisely how our reproducibility protocol was structured. For each fetus, the intra- and interobserver analyses were always performed on the same 5-s four-chamber view cine loop, acquired once and then exported for offline analysis. For intraobserver variability, each operator analyzed this cine loop twice on separate occasions. For interobserver variability, both operators worked on that same cine loop, with the second operator blinded to the results of the first.

Where our approach differs from what Dr. DeVore describes in his comment is that we did not force the operators to use an identical end-diastolic frame or the same cardiac cycle within that cine loop. For the 4CV GSI measurements, each operator selected an end-diastolic frame that they considered to be suitable. For FetalHQ ventricular measurements, the cardiac cycle within the cine loop was chosen by the operator after placing the M-mode line rather than being pre-specified and fixed across all readings. As Dr. DeVore correctly inferred, this means that the exact frame and cycle could differ between readings, although they always came from the same underlying cine loop.

This was not an oversight but a conscious design choice. Our primary aim was to evaluate how FetalHQ behaves in real-world clinical conditions, in the hands of experienced but non-developer users performing routine mid-trimester examinations. In daily practice, the sonographer acquires an acceptable cine loop, selects what appears to be a good representative cycle, traces the endocardium, and moves on. They do not typically mark a given cycle in advance and then force every subsequent analysis to use that exact cycle and frame.

By allowing operators to choose the cycle within the cine loop and to re-position the M-mode line, we deliberately allowed several sources of variability to be expressed: frame/cycle selection, subtle differences in line placement and, above all, endocardial contouring. We fully agree that a strict same-frame same-cycle protocol would have produced higher ICC values and a “cleaner” picture of pure measurement repeatability on a fixed image. Our intention, however, was to capture a more conservative and clinically realistic estimate of what a typical user can expect when implementing FetalHQ in everyday practice.

In other words, our ICC values reflect something slightly different from the scenario Dr. DeVore describes: not only the reproducibility of the software itself when everything is standardized but also the variability introduced by the way clinicians actually work with cine loops at the workstation. We tried to make this point in the Discussion, but we agree that the distinction between an “ideal experimental” setting and a “real-world” setting deserves to be spelled out more explicitly.

We do not see these approaches as competing. On the contrary, we think they are complementary. A same-frame same-cycle study, such as Dr. DeVore outlines, is the best way to document the upper limit of what FetalHQ can achieve under optimal tightly controlled conditions. Our study was designed as a pragmatic independent evaluation of how the method performs when used exactly as it would be in a busy obstetric unit, following consensus guidance but without extra experimental constraints. Ideally, future work should contain both elements side by side: a strictly standardized reproducibility protocol and a parallel arm reflecting routine practice.

We would also like to explicitly acknowledge Dr. DeVore’s central role in the development and promotion of FetalHQ [2]. Our group has learned this method from his original publications, from the consensus documents and from his Voluson Club presentations and online educational material [3]. When we set up our local protocol and training, Dr. DeVore’s work was our main reference. It is therefore entirely natural that his perspective, as one of the originators and key advocates of the method, emphasizes what can be achieved under optimized conditions, while our perspective, as independent users in a general tertiary unit, focuses on what happens when the same tool is exposed to the full variability of routine clinical work. Some divergence in emphasis between these two vantage points is, in our view, not only understandable but also valuable.

Regarding Dr. DeVore’s comment on the use of QUIVER to improve endocardial border delineation, at the time of our data acquisition, this option was not available on our system, so we relied on the standard FetalHQ workflow. We agree that better assistance in defining the endocardial border is likely to reduce one of the main sources of variability that we observed, and we consider this to be an important direction for further refinement of the method.

In summary, we confirm that our analyses were based on the same cine loops but did not enforce identical frames or cycles, and that this was intentional in order to mirror real-life use. We agree that a strict same-frame same-cycle protocol would give complementary and probably higher reproducibility estimates under ideal conditions. We are sincerely grateful for Dr. DeVore’s detailed comments and for the opportunity to clarify these aspects. We hope that combining the perspective of the method’s originator with independent real-world data will help to further develop acquisition, training and interpretation standards for FetalHQ.
